# Conflicting Effects of Methylglyoxal and Potential Significance of miRNAs for Seizure Treatment

**DOI:** 10.3389/fnmol.2018.00070

**Published:** 2018-03-05

**Authors:** Hua Tao, Xu Zhou, Bin Zhao, Keshen Li

**Affiliations:** ^1^Department of Neurology, Affiliated Hospital of Guangdong Medical University, Zhanjiang, China; ^2^Guangdong Key Laboratory of Age-Related Cardiac and Cerebral Diseases, Affiliated Hospital of Guangdong Medical University, Zhanjiang, China; ^3^Clinical Research Center, Affiliated Hospital of Guangdong Medical University, Zhanjiang, China; ^4^Institute of Neurology, Affiliated Hospital of Guangdong Medical University, Zhanjiang, China; ^5^Stroke Center, Neurology & Neurosurgery Division, Clinical Medicine Research Institute & the First Affiliated Hospital, Jinan University, Guangzhou, China

**Keywords:** methylglyoxal, glyoxalase I, receptor of advanced glycation end products, epilepsy, miRNAs

## Abstract

According to an update from the World Health Organization, approximately 50 million people worldwide suffer from epilepsy, and nearly one-third of these individuals are resistant to the currently available antiepileptic drugs, which has resulted in an insistent pursuit of novel strategies for seizure treatment. Recently, methylglyoxal (MG) was demonstrated to serve as a partial agonist of the gamma-aminobutyric acid type A (GABA_A_) receptor and to play an inhibitory role in epileptic activities. However, MG is also a substrate in the generation of advanced glycation end products (AGEs) that function by activating the receptor of AGEs (RAGE). The AGE/RAGE axis is responsible for the transduction of inflammatory cascades and appears to be an adverse pathway in epilepsy. This study systematically reviewed the significance of GABAergic MG, glyoxalase I (GLO1; responsible for enzymatic catalysis of MG cleavage) and downstream RAGE signaling in epilepsy. This work also discussed the potential of miRNAs that target multiple mRNAs and introduced a preliminary scheme for screening and validating miRNA candidates with the goal of reconciling the conflicting effects of MG for the future development of seizure treatments.

## Introduction

Glucose is the most common form of carbohydrate and acts as an essential substrate for brain energy metabolism via glycolysis and subsequent aerobic respiration. Many intermediate metabolites appear during the glycolysis process, particularly methylglyoxal (MG), which is converted from dihydroxyacetone phosphate by MG synthase (Cooper and Anderson, 1970; Cooper, 1974; Tsai and Gracy, 1976). MG has been demonstrated to be a partial agonist of the gamma-aminobutyric acid type A receptor (GABA_A_) and plays an inhibitory role in epileptic activities (Distler et al., 2013). Paradoxically, clinical observations have indicated that carbohydrate-enriched food and drink are precipitating factors of epileptic seizures, and the antiepileptic effects of a ketogenic diet (which comprises high fat, adequate protein and low carbohydrate contents) have been confirmed in children with intractable epilepsy (Kossoff, 2004; Freitas et al., 2007; Luat et al., 2016). Therefore, other substances metabolized from carbohydrates most likely overwhelm the antiepileptic role of MG and shift the balance toward epileptic seizures.

Remarkably, MG itself is an important substrate in the generation of advanced glycation end products (AGEs; Schleicher et al., 2001; Salahuddin et al., 2014). Studies have demonstrated that AGEs are involved in neuropsychiatric and inflammatory diseases through activation of the receptor of AGEs (RAGE; Chuah et al., 2013; Kouidrat et al., 2013, 2015; Fu J. et al., 2017). Epilepsy is characterized by spontaneous synchronized discharge as well as hyperperfusion and metabolic abnormalities of epileptogenic foci during epileptic seizures (Ho et al., 1996; Lu et al., 2006; Bansal et al., 2016; Zhu et al., 2017). Moreover, over the past decade, brain inflammation has been considered a novel mechanism of epilepsy (Dedeurwaerdere et al., 2012; Amhaoul et al., 2014, 2015; Wilcox and Vezzani, 2014). Based on this evidence, the AGE/RAGE axis appears to be an adverse signal transduction pathway for epileptic seizures. This study systematically reviewed the significance of GABAergic MG, glyoxalase I (GLO1), which is responsible for the enzymatic catalysis of MG cleavage, and downstream RAGE signaling in epilepsy and discussed the potential of miRNAs with the goal of establishing groundwork for future research aiming to reconcile the conflicting effects of MG on epileptic seizures.

## GABAergic MG in Epilepsy

### The GABA_A_ Receptor and Its Relevant Ligands

GABA receptors are classified based on their sensitivity to different agonists and antagonists as follows: fast-acting ionotropic GABA_A_ and GABA_C_ receptors and the slower-acting metabotropic GABA_B_ receptor (Bormann, 2000). Among these three subtypes, the GABA_A_ receptor has the most predominant distribution in the brain and has been estimated to be present in 20% to 50% of brain synapses (Nutt and Malizia, 2001). In the brain, neuronal discharges depend on the balance between excitatory glutamatergic and inhibitory GABAergic activities; thus, relative attenuation of GABA_A_ receptor activities is often implicated in a series of excitatory diseases, including anxiety disorders and epilepsy (Sajdyk et al., 2008; Nuss, 2015; Shetty and Upadhya, 2016). GABA is the most abundant inhibitory transmitter in the central nervous system and opens Cl^−^ channels by binding to the GABA_A_ receptor. Notably, benzodiazepines (BDZs) are agonists of the receptor that not only directly open the channel but also augment the capacity of GABA by inducing a global conformational rearrangement of the GABA_A_ receptor (Nutt and Malizia, 2001; Gielen et al., 2012). Based on their key roles in GABAergic activities, BDZs are widely prescribed clinically to treat epileptic seizures (Ochoa and Kilgo, 2016).

### MG Acts as a Partial Agonist of the GABA_A_ Receptor

Distler et al. (2012) observed that the administration of 100 μM MG to hippocampal neurons (HNs) evoked inward Cl^−^ currents by activating the GABA_A_ receptor and that approximately one-third of the magnitude of the inward currents was evoked by 100 μM GABA in HNs. Conversely, the magnitude evoked by the co-application of GABA and MG was approximately two-thirds the magnitude of the inward currents evoked by GABA alone. Based on these *in vitro* findings, we speculate that the roles of GABA and MG in GABAergic activities are competitive instead of additive, implying that both compounds act on the same binding site on the GABA_A_ receptor. Indeed, the Cl^−^ currents evoked by the application of MG to HNs are blocked by the GABA_A_-specific antagonist SR-95531; thus, MG is considered a partial agonist of the GABA_A_ receptor (Distler et al., 2012). Similar to GABA, the MG-evoked Cl^−^ inward currents in HNs are also augmented by the co-application of BDZs (Distler et al., 2012).

As mentioned above, MG is a partial agonist of the GABA_A_ receptor and competitively hampers the GABA-evoked Cl^−^ inward currents that play a key role in increasing the threshold of neuronal discharge. Interestingly, the MG and GABA distributions in the brain do not completely overlap but are complementary to some extent *in vivo*. Previous studies have shown that the GABA concentration in the synaptic cleft peak was in the millimolar range (Farrant and Nusser, 2005) but was extremely low in the extrasynaptic space (less than micromolar; Vithlani et al., 2011). In contrast, MG can be secreted into the extracellular space, and a concentration of 5 μM has been measured in the mouse brain (Distler et al., 2012). Compared with the millimolar concentration of GABA in the synaptic cleft, the micromolar concentration of MG exerts a negligible competitively inhibitory effect on the GABA_A_ receptor, but MG is likely dominant in the extrasynaptic space, where the GABA concentrations are in the sub-micromolar range. Thus, MG might be relevant in inhibiting neuronal discharge through extrasynaptic GABA_A_ receptors. Importantly, the concentration required to achieved the 50% maximal effect (EC_50_) of MG in HNs is 9.5 ± 0.9 μM, whereas the physiological concentration of MG in the rodent brain is 5 μm (Distler et al., 2012); thus, a twofold up-regulation of MG is on the linear segment of the concentration response curve in which a small change in concentration elicits profound effects upon postsynaptic discharge.

More than 30 years ago, GABA and its analogs, such as vigabatrin, were developed to treat epileptic seizures (Gram et al., 1985; Loiseau et al., 1986). However, the use of therapies as seizure treatments is relatively restricted due to many challenges. Principally, GABA binds to all GABA receptors, including the GABA_B_ and GABA_C_ receptors, and this binding elicits a series of additional side effects involved in the activation of the GABA_B_ and GABA_C_ receptors. Moreover, GABA is a fundamental inhibitory neurotransmitter that is necessary for the physiological balance of brain activities; thus, crude and direct interventions for GABA signaling usually result in neuropsychiatric complications, such as impaired concentration, mental decline and depression. GABA has been prescribed at high doses in clinical practice to overcome the blood-brain barrier and increase the amount of GABA in the brain, but long-term administration results in down-regulation of the GABA_A_ receptor and consequent loss of the anti-seizure effects of this treatment. In contrast to GABA, MG does not activate neuronal GABA_B_ receptors, and an effect of MG on the GABA_C_ receptor has not been reported (McMurray et al., 2014). In cooperation with the function of GABA at synaptic GABA_A_ receptors, small changes in MG at doses above 10 μM efficiently strengthen the inhibitory tone (Distler et al., 2012); therefore, MG is a promising compound for modulating the GABA_A_ receptor and related excitatory diseases.

### Antiepileptic Effect of MG

To investigate whether MG prevents or attenuates epileptic seizures, MG was administered before the addition of picrotoxin, which is a GABA_A_ receptor antagonist that can induce seizures in mice (Fisher, 1989; Distler et al., 2013). The MG pretreatment attenuated the generalized convulsions induced by picrotoxin in a dose-dependent manner. Specifically, MG treatment delayed seizure onset, reduced the seizure duration and decreased the percentage of animals that experienced generalized convulsions from the behavioral perspective. Moreover, the antiepileptic effects of MG were examined in a mechanistically distinct epilepsy model established using pilocarpine, which is a muscarinic cholinergic agonist (Curia et al., 2008; Distler et al., 2013). Consistent with the effects observed in the picrotoxin induction model, MG pretreatment delayed acute seizure onset, decreased the seizure duration and reduced the highest seizure stage in a dose-dependent manner. In addition, the role of MG in ongoing seizures was explored by administering MG after pilocarpine administration, and the results showed that animals administered MG spent less time in partial status epilepticus (continuous tremor/clonic seizures of the body and tail while retaining posture; Winawer et al., 2011; Distler et al., 2013). These findings strongly support the potential of MG as a seizure treatment.

## GLO1 Enzyme Activity in Epilepsy

### GLO1 in the Glyoxalase System

The glyoxalase system is responsible for clearing MG in humans and mainly functions as follows. MG combines with the thiol group of reduced glutathione (GSH) to generate the reversible product D-lactoylglutathione. The GLO1 enzyme catalyzes the irreversible transformation of D-lactoylglutathione into S-D-lactoylglutathione, which is further metabolized into nontoxic D-lactate, and GSH is recycled by glyoxalase II (Thornalley, 1990). GLO1 acts as the rate-limiting enzyme in the glyoxalase system and is thus considered a key target to regulate the MG concentration and the pathological accumulation of its downstream AGEs. In other words, based on its enzymatic catalysis of MG, GLO1 likely plays an important role in MG- and AGE-related diseases, and the interest in the potential of GLO1 modulation as a seizure treatment has increased recently (Distler and Palmer, 2012).

### Modulation of GLO1 in Epileptic Seizures

According to previous studies, S-substituted glutathione is a pharmacologic inhibitor of GLO1, and its presence can increase the MG concentration in the brain by approximately 20% (Vince et al., 1971; Thornalley et al., 1996; Distler et al., 2013). Based on this observation, S-substituted glutathione was administered before pilocarpine administration to examine the role of GLO1 in epileptic seizures. Mice pretreated with S-substituted glutathione had a shorter seizure duration than mice pretreated with vehicle (Distler et al., 2013). Moreover, recombinant inbred strains of C57BL/6J and DBA/2J mice (BXD) that inherited a genomic duplication of GLO1 showed an approximately twofold increase in GLO1 expression and an association with increased susceptibility to epileptic seizures at high atmospheric pressure (McCall and Frierson, 1981; Plomin et al., 1991; Distler et al., 2013). Notably, transgenic mice overexpressing GLO1 were also employed to identify the direct effect of GLO1; these mice presented a reduced MG concentration in the brain and increased seizure severity (Distler et al., 2013). In addition, GLO1 polymorphisms are significantly related to epilepsy. In particular, the variation at rs1049346 is potentially useful for assessing the risk of late-onset and drug-resistant epilepsy (Tao et al., 2016). These findings suggest that inhibiting the enzymatic activity of GLO1 and then reducing the clearance of GABAergic MG might be a novel approach for improving epileptic seizures.

## Downstream Rage in Epilepsy

### RAGE in the AGE/RAGE Axis

In addition to acting as a partial agonist of the GABA_A_ receptor, MG rapidly initiates protein glycation via a nucleophilic addition reaction with the free amino group of proteins as well as phospholipids and nucleic acids. This reaction is reversible, but its Schiff base product can also be rearranged to form ketoamine or Amadori products over several days. The Amadori products generate irreversible cross-linkages between adjacent proteins through a series of dehydrations and rearrangements to form AGEs (Schleicher et al., 2001; Salahuddin et al., 2014). In human tissues, AGEs primarily function by activating their membrane receptor RAGE (Chuah et al., 2013; Kouidrat et al., 2013, 2015; Fu J. et al., 2017). AGEs are classified into at least six distinct types based on their origins, including glucose, other carbohydrates, such as glyceraldehyde, and α-dicarbonyls, such as MG, glycolaldehyde, glyoxal, and 3-deoxyglucosone (Takeuchi et al., 2004). Due to the heterogenicity of their origins, AGEs are a poor target for phenotypic and mechanic research, and RAGE is naturally regarded as the focus of the AGE/RAGE axis to explore the pathological significance of AGEs in human diseases.

### RAGE in Inflammatory Diseases

RAGE is a transmembrane protein member of the immunoglobulin superfamily (Schmidt and Stern, 2000) that comprises three immunoglobulin-like domains in the extracellular N-terminal segment, one transmembrane region and a short cytoplasmic C-terminal region; thus, its significance in inflammatory diseases is easily assumed. In fact, RAGE has been demonstrated to mediate a cascade of inflammatory signals, and its interactions with extracellular ligands, such as AGEs, induce the nuclear translocation of nuclear factor kappa B (NF-κB), which promotes the expression of cytokines, chemokines, and adhesion molecules (Chuah et al., 2013). Moreover, RAGE cooperatively signals with Toll-like receptors (TLRs), which function as essential mediators to trigger inflammatory cascades (Mazarati et al., 2011; Chen et al., 2015). In addition, RAGE has been implicated in a wide range of inflammatory diseases, including diabetic complications and atherosclerosis and is critical for the inflammatory response in brain diseases (Ramasamy et al., 2005; Chuah et al., 2013; Singh et al., 2014).

### Inflammatory Mechanism in Epilepsy

Epilepsy is a heterogeneous group of neurological disorders that shares a common manifestation. Excessively abnormal synchronous discharge and alterations of neuronal activities are the primary focuses of epilepsy research. As a result, abnormalities related to ion channels, neurotransmitters and synaptic remodeling were historically considered the most important mechanisms of epilepsy, but this neurocentric emphasis did not completely address issues implicated with the initiation and aggregation of epileptic seizures. In recent decades, brain inflammation was found to be related to drug-resistant epilepsy due to diverse pathological etiologies (Vezzani et al., 2011). Proinflammatory mediators, reactive astrocytosis and activated microglia were observed in the resected hippocampi of patients with temporal lobe epilepsy, but none of these pathologies were present in specimens obtained from healthy control tissues (Crespel et al., 2002). Moreover, lipopolysaccharide, which is a classic inducer of inflammation, lowered the seizure threshold and increased spike-and-wave discharges in a rat model of absence seizures (Kovács et al., 2006). Furthermore, seizure induction can trigger the rapid induction of inflammatory mediators in brain regions prone to the onset and propagation of seizure activity. Importantly, anti-inflammatory and immune therapies, such as adrenocorticotropic hormone, corticosteroids, plasmapheresis and intravenous immunoglobulin have been successfully used to treat epilepsy with varying levels of success (Vezzani et al., 2011). This evidence suggests a positive feedback mechanism between epilepsy and inflammation.

### Inflammatory Involvement of RAGE in Epilepsy

Many lines of evidence highly support the inflammatory involvement of RAGE in epileptic seizures. Rasmussen encephalitis (RE) is a rare brain disorder characterized by unihemispheric inflammation, progressive neurological deficits and intractable focal epilepsy. Interestingly, increasing levels of RAGE expression were observed in surgically resected epileptic cortical specimens from patients with RE (Luan et al., 2016), implying the involvement of RAGE in inflammatory RE. High-mobility group box 1 (HMGB1), which is another endogenous ligand of RAGE, contributes to the overexpression of P-glycoprotein (P-gp) in mouse epileptic brain tissues by activating TLR4/RAGE receptors and the downstream transcription factor NF-κB in brain microvascular endothelial cells (Chen et al., 2015). Because P-gp is encoded by a gene involved in multidrug resistance and NF-κB plays a key role in the inflammatory response, RAGE has been proposed as a critical molecule responsible for inflammation and drug resistance in epilepsy. In addition, HMGB1 was ubiquitously expressed in glial and neuronal cells in control and pathological cortex specimens, but RAGE expression was only observed in cortical dysplasia where epileptic seizures manifested (Zurolo et al., 2011). Compared with its ligand HMGB1, RAGE activity likely plays a pivotal role in the transduction of inflammatory signals. Recently, a dominant inheritance model of the G82S locus in RAGE was observed to be protective against drug-resistant epilepsy (Guo et al., 2016), providing genetic support for the involvement of RAGE in epileptic seizures.

## Dual Regulation by miRNAs in Epilepsy

### Conflicting Effects of MG

**Figure 1:**
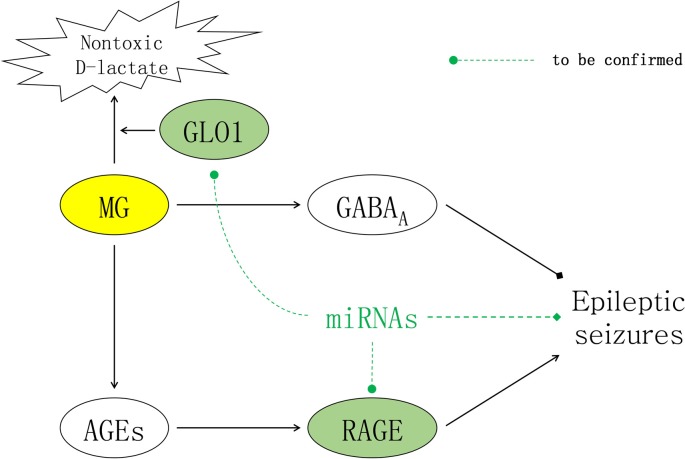
Contradictory effects of methylglyoxal (MG) for seizure treatment. In the brain, MG inhibits epileptic seizures by activating extrasynaptic gamma-aminobutyric acid type A (GABA_A_) receptors. The inflammatory effects of downstream advanced glycation end products (AGEs) are mediated by their receptor receptor of AGEs (RAGE), which can induce epileptic seizures. As illustrated above, miRNAs are molecules with the potential to reconcile the conflicting effects of MG and alleviate epileptic seizures by synergistically targeting both glyoxalase I (GLO1) to increase the GABAergic effects of MG and RAGE to inhibit the inflammatory effects of AGEs.

As mentioned above, epileptic seizures were theoretically alleviated in animal epilepsy models in which GLO1 activity was reduced, resulting in insufficient clearage of MG. However, AGEs simultaneously accumulate in response to excessive MG concentrations, and this accumulation activates RAGE and results in inflammatory cascades that aggravate epileptic seizures. Due to the adverse effects of AGEs, the prospect of modulating the glyoxalase system for achieving seizure control has historically been undervalued. Notably, AGEs function by binding to RAGE, and combined inhibition of GLO1 and RAGE might be an approach to reconcile the conflicting effects of MG on epileptic activities by synergistically increasing the GABAergic effects of MG while simultaneously inhibiting the inflammatory effects of AGEs (Figure 1).

### miRNAs With Multiple Targets

Non-coding RNAs were once classified as transcriptional noise, but these molecules have attracted increasing attention due to their regulatory effects on protein-coding genes. Among non-coding RNAs, miRNAs are a specific class of small non-coding molecules that negatively regulate gene expression by complementarily binding to sequences in the 3′ untranslated regions (UTRs) of target messenger RNAs (mRNAs) in eukaryotes; these interactions result in either direct cleavage of the mRNA or translational inhibition. Based on the base-pairing principle, each miRNA usually regulates more than one mRNA that possesses a complementary sequence in its 3′ UTR. Thus, miRNAs are regarded as promising candidates to regulate multiple targets at the post-transcriptional level.

To date, dual regulation of miRNAs has been demonstrated in many pathological conditions, such as ovarian injury and apoptosis (Du et al., 2017; Fu X. et al., 2017; Chang et al., 2018), which raised a profound question as to whether miRNAs could improve epilepsy by targeting both GLO1 and RAGE.

### Potential miRNAs Targeting Both GLO1 and RAGE

As mentioned above, miRNAs function at the post-transcriptional level and have been demonstrated to be a promising approach for fine-tuning the excessive expression of their target genes in several biological processes (Rahkonen et al., 2016; Roy, 2016; Su et al., 2016). In addition, intranasal delivery of miRNA mimics and inhibitors, which can bypass the blood-brain barrier to deliver drugs into the brain, has been used recently to treat several neurological diseases (Ma et al., 2016; Lee et al., 2017; Tao et al., 2017). However, miRNAs have not been reported to target both GLO1 and RAGE, and their roles in epilepsy remain unknown. Regardless, bioinformatics analyses can be used to screen miRNA candidates targeting both GLO1 and RAGE. If any candidate is confirmed using a dual-luciferase report gene system, the potential of using miRNAs for seizure treatment warrants investigation through future experiments.

## Conclusion

This study systematically reviewed the significance of MG, GLO1 and RAGE in epilepsy, discussed the potential of miRNAs and introduced a preliminary scheme to screen and validate miRNA candidates with the goal of reconciling the conflicting effects of MG for the future development for seizure treatments.

## Author Contributions

HT and XZ wrote the manuscript. BZ and KL reviewed the topic and established a basic framework for the manuscript.

## Conflict of Interest Statement

The authors declare that the research was conducted in the absence of any commercial or financial relationships that could be construed as a potential conflict of interest.
